# Analysis of graduating nursing students’ moral courage in six European countries

**DOI:** 10.1177/0969733020956374

**Published:** 2020-10-29

**Authors:** Sanna Koskinen, Elina Pajakoski, Pilar Fuster, Brynja Ingadottir, Eliisa Löyttyniemi, Olivia Numminen, Leena Salminen, P Anne Scott, Juliane Stubner, Marija Truš, Helena Leino-Kilpi, On behalf of ProCompNurse Consortium

**Affiliations:** 8058 University of Turku, Finland; 88179 International University of Catalonia, Spain; 63541 University of Iceland and Landspitali University Hospital, Iceland; 8058 University of Turku, Finland; 8799 National University of Ireland, Ireland; 543635 Martin Luther University Halle-Wittenberg, Germany; 87241 Klaipeda University, Lithuania; 8058 University of Turku and Turku University Hospital, Finland

**Keywords:** Ethical competence, graduating nursing student, international survey, moral courage, nursing education

## Abstract

**Background:**

Moral courage is defined as courage to act according to one’s own ethical values and principles even at the risk of negative consequences for the individual. In a complex nursing practice, ethical considerations are integral. Moral courage is needed throughout nurses’ career.

**Aim:**

To analyse graduating nursing students’ moral courage and the factors associated with it in six European countries.

**Research design:**

A cross-sectional design, using a structured questionnaire, as part of a larger international ProCompNurse study. In the questionnaire, moral courage was assessed with a single question (visual analogue scale 0–100), the questionnaire also covered several background variables.

**Participants and research context:**

The sample comprised graduating nursing students (n = 1796) from all participating countries. To get a comprehensive view about graduating nursing students’ moral courage, the views of nurse managers (n = 538) and patients (n = 1327) from the same units in which the graduating nursing students practised were also explored, with parallel questionnaires.

**Ethical considerations:**

Ethical approvals and research permissions were obtained according to national standards in every country and all participants gave their informed consent.

**Results:**

The mean of graduating nursing students’ self-assessed moral courage was 77.8 (standard deviation 17.0; on a 0–100 scale), with statistically significant differences between countries. Higher moral courage was associated with many factors, especially the level of professional competence. The managers assessed the graduating nursing students’ moral courage lower (66.5; standard deviation 18.4) and the patients slightly higher (80.6; standard deviation 19.4) than the graduating nursing students themselves.

**Discussion and conclusions:**

In all countries, the graduating nursing students’ moral courage was assessed as rather high, with differences between countries and populations. These differences and associations between moral courage and ethics education require further research.

## Introduction

Moral courage has been described as a complex, multidimensional concept,^1–3^ having its roots in virtue ethics.^4^ Moral courage can exist and develop only when an individual aims for it.^3^ It is said to be a part of nurses’ ethical competence.^2,3,5^ According to the competence criteria for registered nurses (RN) in the European Union, a defined level of ethical competence and knowledge of professional ethics is required to ensure patient safety and quality of care.^6,7^ Moral courage is needed in nursing practice for promoting ethical, humanised care when facing ethical conflicts and for promoting patients’ rights.^8–11^ Therefore, it is important to develop moral courage already during nursing education.^12–14^

Moral courage is described as the courage needed for defending one’s own moral principles, even at the risk of negative outcomes for the individual.^2,3,11,15,16^ Moral courage always requires profound consideration between various options, including the other viewpoints as well, and finally, decision-making.^16^ Moral courage is described as an exalted virtue in philosophy,^16^ psychology,^15^ and nursing^2,3^ that can be learned and developed.^2,17^

Moral courage has been studied from the perspective of nursing students also earlier, mainly in the 2000s.^5,9,12,14,17^ It has been shown that students feel an obligation to act as patients’ advocates,^12,17^ and for this, they need moral courage.^9,14,17^ However, students sometimes seem to lack courage.^9,14,17,18^

Moral sensitivity, which is the ability to recognise moral situations,^19–21^ has been identified as a prerequisite for acting with moral courage. Students seem to have moral sensitivity, and education has a positive impact on its development.^21^ Elements of nursing students’ moral courage can also be found in relation to whistleblowing,^4,18,22^ moral integrity^23,24^ and being a professional nurse.^2,25,26^ Moral courage has also been analysed in relation to moral distress,^13,27–29^ referring to situations when a person is not able to act according to ethical principles.^29^ Moral courage can reduce moral distress,^2^ and students need support to confront moral distress and develop their moral courage.^29^ The positive consequences resulting from moral courage refer to personal and professional growth, empowerment and calmness observed as professional care.^2,3^ Moral courage also supports nurses in their career path^3^ and development of the profession and work environment.^30^

Various factors support moral courage. In social communities, moral courage can spread, enabling more people to act morally courageously.^31^ Support has been identified both in nursing education^12,17^ and in nursing practice.^3,8^ In education, supporting factors include the learning of ethics which can strengthen nurses’ behaviour in ethical conflicts as a prerequisite of moral courage.^12,13^ Moreover, a respectful student–mentor relationship during clinical practicums,^9,12^ participation in discussion on ethics, students willingness to act as patients’ advocate^9,17^ and educational interventions^14^ seem to be beneficial for students’ moral courage.^9,17^ Moreover, nurse educators can strengthen nursing students’ moral courage by enhancing discussion of ethics and by acting as role models.^12,17^

Nursing students’ moral courage seems to develop gradually along with their ethical competence, the development leading towards independent acting in ethical situations.^17^ In nursing practice, clinical competence,^2,3^ nurses’ behavioural and control beliefs,^32^ a good ethical climate^3,8,30^ and discussion of ethical questions^2^ can strengthen moral courage. Furthermore, nurse managers can promote ethical climate by encouraging discussion and collaboration between professionals.^8^

Factors inhibiting nursing students’ moral courage have also been identified. These include lack of professional competence,^17^ being low in the professional hierarchy and consequently having feelings of powerlessness,^9^ lack of support from clinical supervisors^12^ and difficulty speaking up when facing poor care.^9,17^

Research on nursing students’ moral courage has been conducted in different contexts, such as clinical practicums,^9^ community workshops^14^ and nursing schools.^17^ The previous studies have been either descriptive small-scale studies^9,17^ or conducted with national samples.^14^ Consequently, studies with larger samples and varying designs are needed. Especially, there is a lack of international, cross-cultural research assessing graduating nursing students’ moral courage.

## Aim and research questions

The aim of this study was to analyse graduating nursing students’ (GNS) moral courage and the factors associated with it in six European countries. The following research questions were addressed:

1. What is the GNSs’ self-assessed moral courage?
2. What factors are associated with the GNSs’ self-assessed moral courage, if any?
3. What is the relationship between the self-assessed moral courage of GNSs and the assessments of nurse managers and patients?

The goal of this study was to add new knowledge to the discussion about GNSs’ moral courage by combining the assessments of students, nurse managers (hereafter, managers) and patients. For nursing education, the knowledge gained from this study can contribute to the harmonisation and development of ethics education in Europe.^33^ For nursing management and practice, the acquired knowledge can strengthen the provision of support for nurses’ ethical competence.^34^ Moreover, managers often encounter ethical problems related to nursing staff,^35^ and for early career nurses in particular, managers are the key persons in supporting the transition to practice as well as overseeing nurses’ competence development,^36^ including ethics. For patient care, patients experiencing GNSs’ moral courage while receiving care are among the legitimate stakeholders to provide their evaluations. By participating in research, patients’ perspective can be taken into account in education aimed at healthcare professionals.^37^

## Research design

This study applied a cross-sectional survey design, the target populations including GNSs, managers and patients in six European countries. This study is part of a large international prospective longitudinal study entitled Professional Competence in Nursing (ProCompNurse). These data, in the first phase of the longitudinal study, have been collected at the end of GNSs’ nursing education, illustrating the first step in the nursing career.

## Participants and research context

The primary study population consisted of GNSs from southern (Spain), northern (Finland, Iceland), central (Germany, Lithuania) and western (Ireland) Europe. The inclusion criteria for the GNSs were that a student (1) was studying in a programme leading to the qualification of a RN and (2) was close to graduation.

The graduating nursing students sample size was calculated for the research project based on the Nurse Competence Scale (NCS).^38^ The relevant mean difference was regarded as five points and standard deviation was 15.7.^39^ With statistical power of 80% and significance level of 0.05 (two-tailed), the minimum sample size in each country was 156 respondents. However, the desired sample size was increased to 500 due to probable loss in the follow-up phase. It was acknowledged that the different population sizes in each country set limitations for this goal.

In addition to GNSs, managers and patients were recruited in the units in which the GNSs currently practised. By adding these two groups, the aim was to reach a more comprehensive view^40,41^ of GNSs’ moral courage. The basic understanding was that managers would know about the moral courage of GNSs due to their role as recruiting nurses throughout the whole career. Patients, on the other hand, were assumed to be those experiencing the GNSs’ moral courage during their practicum. Included in the study were managers who (1) had a nurse background, (2) were in a managerial position and had daily or almost daily contact with the clinical nursing staff and (3) were responsible for or contributed to the recruitment of nursing workforce. Patients eligible for the study had to be (1) at least 18 years of age, (2) able to give an informed consent and (3) able to respond to the questionnaire based on their language competence and overall health condition.

Convenience sampling was used for all populations. Geographical representativeness within countries was taken into account when applicable. Data were collected from May 2018 until March 2019 from hospitals mainly providing tertiary level care and from several educational institutions responsible for the education of RNs (Table 1).

**Table 1.** The numbers of surveyed organisations and respondents.

Country	Surveyed organisations		
	GNSs n, response rate	Managers n, response rate	Patients n
Finland	12 Universities of applied sciences 514, 37%	5 University hospitals 1 Hospital 112, 33%	5 University hospitals 2 Hospitals 270
Germany	12 Nursing schools of university hospitals 2 Nursing schools of other hospitals 304, 55%	6 University hospitals 1 Hospital 92, 97%	6 University hospitals 2 Hospitals 135
Iceland	2 Universities 64, 55%	1 University hospital 30, 75%	1 University hospital 137
Ireland	6 Universities 399, 88%	6 University hospitals 120, 88%	5 University hospitals 299
Lithuania	1 University 5 Colleges 272, 58%	7 Hospitals 85, 71%	1 University hospital 3 Hospital 2 Outpatient departments 263
Spain	5 Universities 243, 36%	4 University hospitals 1 Hospital 99, 66%	4 University hospitals 3 Hospitals 223
Total	45 1796, 49%	32 538, 66%	34 1327

GNS: graduating nursing students.

### Data collection procedure

In the assessment of GNSs’ moral courage, a visual analogue scale (VAS 0–100) derived from the Nurses’ Moral Courage Scale (NMCS; ^©^Numminen)^11^ was used (0 = I never act morally courageously, although the care situation would require it and 100 = I act morally courageously always when care situation requires it). For every population (GNSs, managers, patients), the same definition was presented in the questionnaire: ‘Moral courage is the nurse’s ability to rationally defend professional ethical principles and to act accordingly despite the anticipated or real adverse consequences of such action’ (Table 2).^11^

**Table 2.** The GNSs’ moral courage assessed by themselves, managers and patients.

Population Moral courage	Countries										Model-based mean estimate for populations and (95% CI)
	Total	Finland	Germany	Iceland	Ireland	Lithuania	Spain	Comparison between countries^a^	Comparison between populations^a^	Interaction between countries and populations^a^	
**GNSs, n**	1503	436	277	48	315	255	172	*p* < 0.0001^b^	*p* < 0.0001^c^ *p* < 0.0001^d^ *p* < 0.0001^e^ *p* = 0.0074^f^	*p* = 0.0637^g^	
** Mean (SD)**	77.8 (17.0)	73.9 (17.1)	79.9 (15.8)	76.0 (17.3)	79.7 (16.3)	77.4 (18.8)	82.0 (15.2)				78.7 (77.7; 79.7)
**Managers, n**	493	108	87	26	97	82	93				
** Mean (SD)**	66.5 (18.4)	62.2 (16.3)	64.9 (16.9)	74.6 (17.0)	68.7 (17.8)	68.7 (20.2)	66.7 (20.4)				67.0 (65.3; 68.6)
**Patients, n**	1196	234	118	96	284	257	207				
** Mean (SD)**	80.6 (19.4)	77.4 (18.1)	78.8 (18.5)	84.2 (17.1)	80.4 (20.8)	81.7 (20.7)	82.6 (18.2)				80.9 (79.8; 81.9)

GNS: graduating nursing students; CI: confidence interval; SD: standard deviation.

^a^ Comparisons between populations and countries were performed with two-way analysis of variance (ANOVA).

^b^ Overall differences between the countries.

^c^ Overall population difference.

^d^ Difference between managers and patients.

^e^ Difference between managers and GNSs.

^f^ Difference between GNSs and patients.

^g^ Analysed whether difference in populations differed significantly between countries.

Several individual, educational and value-based background factors potentially associated with moral courage were also asked (Table 3). To collect these factors, either VASs^38,42^ or Likert-type scales were used. The data from GNSs, managers and patients were collected with parallel instruments, with varying sociodemographic background questions. The instruments were translated into national languages through a double translation process^43^ and piloted in every country for evaluation of the understandability and usability of the instruments.

**Table 3.** Associations between GNSs’ background factors and assessments about their moral courage.

Background factor	Model-based mean estimate /slope (B)	95% CI	Univariate *p* value^a^	Multivariable *p* value^a^
Individual factors				
Age (years)	0.152	0.016–0.287	0.0284^*^	
Total length of work experience in health care (months)	0.029	0.002–0.055	0.0332^*^	0.4400
Gender				
Female	78.2	77.1–79.3	0.9235	
Male	78.1	75.5–80.6		
Education level when entering nursing programme				
Upper secondary degree (vocational and/or general)	78.3	76.9–79.7	0.6517	
College-level/post-secondary non-tertiary degree	77.3	75.0–79.5		
Higher education/university degree	79.2	75.7–82.6		
Previous degree in health care				
Yes	80.2	78.0–82.4	0.0384^*^	
No	77.8	76.6–79.0		
Nursing the first study option				
Yes	78.4	77.1–79.6	0.5438	
No	77.8	76.1–79.5		
Nursing career plan for the future				
Yes	79.2	77.9–80.5	0.0060^*^	
No	76.7	75.1–78.2		
Plan to change into another degree in health care				
Never	78.8	77.1–80.4	0.5300	
Fairly seldom	77.3	75.7–78.9		
Fairly often	78.6	76.5–80.8		
Very often	78.3	75.3–81.2		
Plan to change into another degree outside of health care				
Never	78.9	77.4–80.4	0.2846	
Fairly seldom	77.2	75.5–78.9		
Fairly often	77.3	75.0–79.7		
Very often	79.3	76.4–82.3		
Educational factors				
Evaluation of the nursing degree programme				
Very dissatisfied	77.9	72.6–83.2	0.1444	
Dissatisfied	77.1	74.9–79.4		
Satisfied	77.9	76.6–79.1		
Very satisfied	80.8	78.3–83.4		
Level of study achievements				
Very poor	73.0	60.5–85.4	0.0008^*^	0.2532
Poor	73.8	70.1–77.4		
Good	77.8	76.6–79.0		
Excellent	82.1	79.6–84.7		
Evaluation of the supervisory relationship (Likert 1–5)	0.838	−0.126–1.802	0.0882	
Total NCS score (VAS 0–100)	0.436	0.378–0.493	<0.0001^*^	<0.0001^*^
Interaction between moral courage, total NCS score and country			–	
Finland	0.647	0.535–0.759		<0.0001^*^
Germany	0.340	0.174–0.507		
Iceland	0.789	0.385–1.193		
Ireland	0.294	0.156–0.432		
Lithuania	0.235	0.109–0.361		
Spain	0.360	0.191–0.529		
Value-based factors				
Confidence in caring according to the ethical principles (VAS 0–100)	0.540	0.496–0.584	<0.0001^*^	
Evaluation of the nursing profession				
Fully unsatisfied	82.6	77.5–87.8	<0.0001^*^	
To some extent unsatisfied	75.2	72.5–78.0		
To some extent satisfied	76.7	75.2–78.2		
Fully satisfied	80.3	78.8–81.8		
Valuation of nursing in one’s country				
Fully disagree	80.1	77.9–82.4	0.0293^*^	0.2939
Disagree to some extent	76.7	75.1–78.3		
Agree to some extent	78.4	76.8–80.0		
Fully agree	80.4	76.6–84.1		

GNS: graduating nursing students; CI: confidence interval; NCS: Nurse Competence Scale; VAS: visual analogue scale.

^a^ Linear models for studied association between moral courage and background factors were build up first with univariate approach followed by multivariable approach.

*<0.05.

For data collections, each educational institution and hospital named a contact person(s) to collaborate with the researchers. The GNSs’ data were mainly collected in electronic format by distributing a survey link to eligible GNSs to their school email. REDCap electronic data capture software hosted at the University of Turku was used.^44^ Alternatively, paper-and-pencil questionnaires were used if this was preferred by the educational institution. Efforts were made to reach as many eligible GNSs as possible by requesting whether the educational institutions could allocate time for GNSs to answer during class time and sending up to two reminders.

The managers’ data were collected with paper-and-pencil questionnaires; Irish managers also received a parallel electronic survey via REDCap to their work email. Managers returned the questionnaires anonymously in sealed envelopes as agreed locally. Patients were recruited from the same units where the GNSs did their clinical placements. Eligible patients were selected in collaboration with staff nurses or clinical supervisors. Either they or the researcher(s) informed the patients about the study, requested their consent and after that, handed out the questionnaires. Patients returned the questionnaires anonymously in a sealed envelope to their units, from where the questionnaires were delivered to the researchers.

### Statistical analysis

First, comparison between countries for each respondent group (population) was executed with one-way analysis of variance (ANOVA). Second, comparison between GNSs, managers and patients was performed with a two-way ANOVA, in which both the respondent group and the country were handled as categorical explanatory variables. Checking for model assumptions was done from studentised residuals. When the main result was significant, *p* values from pairwise comparisons were corrected using Tukey’s method.

The graduating nursing students data were analysed in more detail. Linear models were used to analyse which explanatory variables were associated with moral courage and whether the association varied between the countries. In these models, the length of work experience in months, valuation of nursing in the country, satisfaction with practicum, overall professional competence level (total NCS score) and all interaction with country were analysed; interaction meant studying whether association between moral courage and, for instance, the length of work experience in months differed between the countries. Non-significant interactions were removed from the final model.

Confidence intervals (CI) of 95% were calculated. Statistical tests were performed as two-sided, with a significance level set at 0.05. The analyses were performed using SAS software, version 9.4 for Windows (SAS Institute Inc., Cary, NC, USA).

## Ethical considerations

Good scientific practice was followed in the study.^45^ The ethical approval for the ProCompNurse project was received from the Ethics Committee of the University of Turku (Statement 62/2017, 11.12.2017) and ethical approvals and research permissions were granted according to national standards in every country. Permissions for translating and using the instruments were received from their copyright holders.

All participants received a cover letter informing them about the voluntariness of participation, confidentiality and their right to withdraw at any stage of the study. Signed consents were requested from the GNSs in every country as contact information was requested due to the follow-up nature of the study.^46^ Depending on national policies, signed consents from managers and patients were requested or the returning of the questionnaire was regarded as a consent to participate. In every country, it was ensured during patient recruitment that patients’ health condition permitted their participation.^47^

## Results

### Participants

The graduating nursing students (n = 1796) were mostly women (n = 1563, 88.0%), and their (n = 1771) mean age was 25.5 years (SD 6.7, range 18–60 years). Two-thirds (n = 1168, 66.4%) had an upper secondary school qualification before entering nursing studies. About one-fifth (n = 349, 19.6%) had a previous degree in health care, and more than half (n = 1079, 60.7%) had working experience in the field (median 18 months, Q1–Q3 7.0–36.0). For two-thirds (n = 1262, 70.9%), nursing had been the first choice of study. Two-thirds (n = 1177, 70.5%) had hardly ever planned to change into another degree programme in health care, and about the same number (n = 1223 74.4%) had hardly ever planned to change into another degree outside of health care. Similar numbers of GNSs had plans for nursing career (n = 1115, 63.2%) and for further studies (n = 1219, 69.1%).

Graduating nursing students (n = 1259, 78.7%) were mostly satisfied with their nursing education, 1494 (93.4%) rating their level of study achievements as good or very good. The overall professional competence level of the GNSs, measured with the NCS, (n = 1686) was 62.2 (SD 14.9) and their (n = 1644) confidence to provide care based on ethical principles of nursing was 77.6 (SD 16.5). GNSs (n = 1598) evaluated the content of the supervisory relationship during the latest clinical practicum as positive (mean 4.0, SD 0.9). The majority of the GNSs were satisfied with belonging to the nursing profession (n = 1341, 85.4%), while less than half (n = 678, 43.4%) felt that the nursing is valued in their country.

The managers (n = 538) were mostly women (n = 480, 89.9%) with a mean age of 46.4 years (SD 10.0 years, range 23–68 years) and had on average 11.7 years of working experience as managers (SD 9.8, range 0–41). The managers were most commonly assistant unit nurse managers (or equivalent; n = 235, 45.0%). As a post-graduate degree, one-third had either a Master’s degree or PhD (n = 149, 30.2%). Two-thirds (n = 309, 61.8%) of the managers thought that nursing is valued in their country.

Just over half of the patients (n = 1327) were women (n = 704, 53.7%) and their mean age was 60 years (SD 16.3 years, range 18–98 years). Two-thirds (n = 802, 62.2%) had a long-term diagnosis. Nearly half of the patients (n = 564, 43.1%) assessed their health status as average (2.96 on 1–5 Likert-type scale). Most patients (n = 922, 72.0%) thought that nursing is valued in their country.

### Graduating nursing students’ moral courage

Graduating nursing students moral courage was assessed both within and between the respondent groups (populations) in every country (Table 2). Within the total sample of GNSs (n = 1503), students self-assessed their moral courage to be 77.8 (range 73.9–82.0; scale 1–100; SD 17.0), and the variation between different countries (73.0–82.0) was statistically significant (*p* < 0.0001). Managers (n = 493) assessed GNSs’ moral courage at 66.5 (SD 18.4); again, the variation between countries (62.2–74.6) was statistically significant (*p* = 0.014). Within the total sample of patients (n = 1196), the GNSs’ moral courage was assessed to be 80.6 (SD 19.4), with statistically significant (*p* = 0.018) variation between countries (77.4–84.2).

Between the respondent groups, there were also variations both within the total sample and between the countries. There was a statistically significant (*p* < 0.0001) difference between the assessments of the respondent groups in all countries, and the differences between the respondent groups were aligned in different countries (*p* = 0.0637). Also, in the assessments of the GNSs and patients, a statistically significant difference (*p* = 0.0074) was found. Patients (80.6; SD 19.4) assessed the GNSs’ moral courage higher than both managers (66.5; SD 18.4) and the GNSs themselves (77.8; SD 17.0) and in all other countries except Germany, where the highest assessment was the GNSs’ own assessment. The managers’ assessment was the lowest both in their total sample and in every country.

### Background factors associated with graduating nursing students self-assessment of their moral courage

Based on univariate analysis, GNSs’ self-assessment of moral courage was positively associated with several background factors (Table 3): being older (*p* = 0.0284), having longer work experience in health care (*p* = 0.0332), having a previous degree in health care (*p* = 0.0384), having nursing career plans (*p* = 0.0060), assessing their study achievements as excellent (*p* = 0.0008) or being dissatisfied with the nursing profession (*p* ≤ .0001). In addition, higher confidence in their ethical principles (*p* ≤ .0001), assessing nursing valued in their country (*p* = 0.0293) and assessing their professional competence at a high level (*p ≤* .0001) were factors positively associated with moral courage.

In order to test the association of individual, educational and value-based background factors with moral courage, the following variables were selected for further analysis: total length of work experience in health care, overall professional competence level (total NCS score), the level of study achievements and the assessment of valuation of nursing in one’s own country.

**Figure 1.**
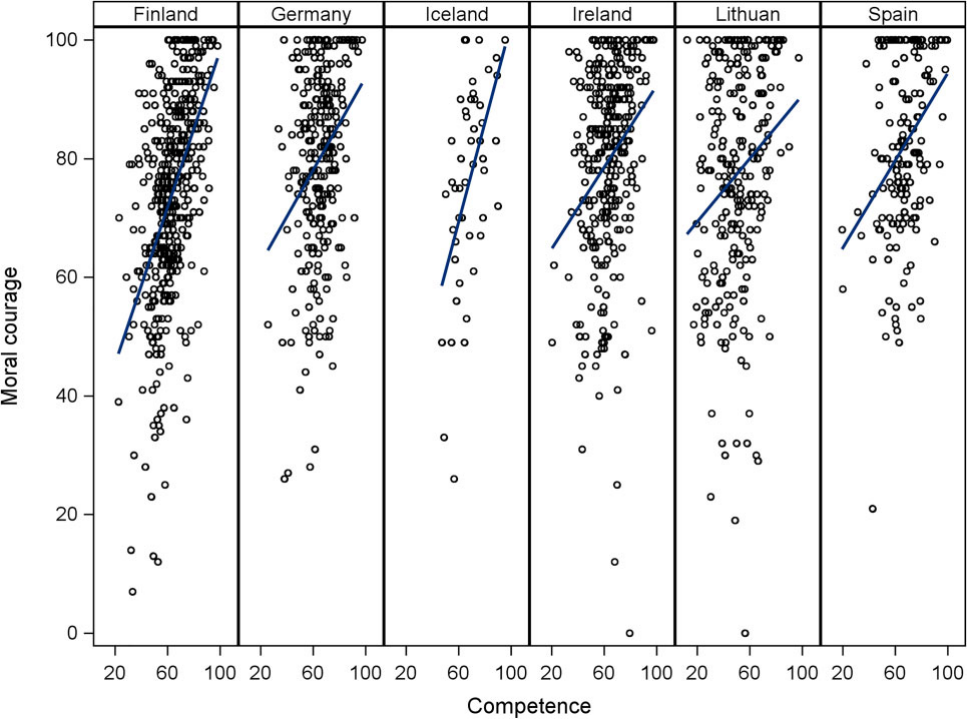
Association between moral courage and NCS separately for each country. Scatter plot together with linear regression line is shown for illustration purposes. Regression line expresses the association between moral courage and NCS.

The final model indicated a high association between the GNSs’ self-assessed moral courage and both NCS score (*p* ≤ .0001) and country (*p* ≤ .0001). Moreover, the factors’ interactions were statistically significant (*p ≤* .0001) and varied between countries; that is, the associations were even more prominent in some countries than in others (Figure 1).

## Discussion

In this article, the goal was to add new knowledge to the discussion of GNSs’ moral courage, combining the assessments of students, managers and patients and analysing these in six European countries. The GNSs’ self-assessed moral courage was rather high, in line with earlier studies,^9,13,17^ leaving less impact for development during the career than has been suggested in earlier research.^2,3^ Notably, however, it is possible that social desirability bias may have influenced the GNSs’ responses.^48^ Due to the ethical nature of nursing, GNSs might feel the obligation to act morally courageously^11^ and thus, may have answered accordingly. Furthermore, the cultural values in each country can have an impact;^49^ however, this was not examined or controlled in this study. Nevertheless, all countries were considered to share the overarching European value-base, making the results comparable.

Graduating nursing students high self-assessments may be a sign of the difficulties of assessing such a complex and abstract concept as moral courage^11^ especially as no attachment to context or case examples were presented. Even if a context or case is presented, individuals can still have different and sometimes very definite views about what is right or wrong; consequently, situations may not be viewed in exactly the same way by all. Moreover, assessment is challenging because courage is always based on judgement, and thus, either extremity – cowardice or foolhardiness – does not demonstrate an individual’s courage.^16,50^ Overall, for anyone assessing moral courage, it would be important to understand the concept while not forgetting to pay attention to contextual and personal factors;^1^ this is most likely the development area for GNSs. In this study, the premise was virtue ethics, but it could also have been normative ethics, where the correct way to act is to act according to legislation, organisational processes and policies as well as ethical codes. Further studies about the concept of moral courage and factors contributing to its actualisation are warranted.

There were statistically significant differences in the self-assessments of GNSs’ moral courage between the participating countries. In earlier research, differences on European level have been indicated in the perceived frequency of respect and human presence^51^ and in nurses’ perceptions of the realisation of autonomy, privacy and informed consent^52^ in nursing. In this study, there may be several reasons behind country differences. The professional roles and responsibilities of both nurses and GNSs in clinical practice may vary between countries^9^ and there are variations in measurable indicators, such as the number of practising nurses per 1000 population (highest in Iceland and Finland, lowest in Spain) or nurse–doctor ratio (highest in Ireland and Finland, lowest in Spain).^53^ However, these factors do not provide straightforward explanations.

In Finland, all respondent groups assessed the GNSs’ moral courage as lowest in comparison to other countries, while the highest scores from managers and patients came from Iceland and the highest self-assessments from GNSs came from Spain. For Finland, the current findings align with the previous ones although they are not fully comparable. Nurses have reported troubled conscience due to occasional inadequacy in providing good care.^54^ In addition, difficulties to act morally courageously have been reported,^55,56^ especially when confronting physicians.^56^ Although these are not unique features globally, they may be signals resulting from the working conditions of Finnish RNs. For instance, it has been found that there are notable differences in the organisation of hospital-based nursing care for RNs even between the Nordic countries which otherwise share many similarities in their health care systems. Compared to their Swedish and Norwegian colleagues, for instance, Finnish RNs face a higher patient workload.^57^ Therefore, it would be relevant to explore this connection between workload issues and moral courage as one possible explanation for the assessments. Nonetheless, nurses’ ethical competence has been indicated to be at an average or high level in Finland.^34,56^

In Iceland, students very frequently work within nursing for years before graduation (a statistically significant difference to other countries shown also in this data set, but not reported here in detail) and thus gain experience, possibly by repeatedly witnessing situations requiring moral courage. Moreover, in a recent international study on missed nursing care, Iceland scored lowest on missed nursing care, while job satisfaction was highest among the Icelandic nurses.^58^ This may indicate that GNSs enter work environments which support them in developing their moral competence. However, issues in missed care have not been investigated from the ethical perspective, and for example, the patients’ perspective in the present studies is very limited.^59^ As for Spain, no explicit reason for the highest score among GNSs can be given; nationally, the finding aligns with a previous Spanish study where GNSs gave more importance to ethical values than experienced nurses,^60^ such as the managers in this study. To sum up, previous research does not show a connection to moral courage for any of the above-mentioned aspects. Thus, there is clearly an overall need for further evidence of these differences between the countries.

In addition, nursing education itself can be one possible explanation for the differences between the countries despite the common European directives guiding education.^6^ Besides differences in nursing education in general,^61^ the teaching of ethics and its aspects like moral courage may also vary. That is, GNSs may not have similar readiness to practise and act in ethical situations as the common directive regulates only that the degree programme should include teaching about ethics but does not address the extent of these studies, for instance.^6^ However, competence in professional/ethical values and practice has also been identified as one of the core competence areas of nurses internationally,^39^ assuming that ethics content is covered in European nursing curricula while demonstrating that ethical/legal principles have also been pointed as one of the core competences and teaching domains for nurse educators.^62^ Based on the results, it is otherwise difficult to identify the connection to the extent of ethics teaching and/or the didactical solutions used. However, the results justify further studies in this area given the joint labour market. Nonetheless, as an educational implication based on the results, it is suggested that ethics education should remain at least at its present level. Moreover, it would be a good idea to pay attention to the teaching of virtue ethics in addition to normative ethics, such as legislation and codes. Nurse educators are also invited to appraise whether the provided teaching enhances students’ ability to reflect their actions in ethically demanding situations.

As for the factors associated with moral courage, the results of this study confirm particularly the association between professional competence and moral courage.^2,3,17^ This is an indication of how important it is to connect the teaching of ethics with the teaching of different parts of professional competence; that is, ethics should be linked to all teaching areas, including the clinical practicum.^63^ Various other background factors were also associated with GNSs’ moral courage. However, there are some inconsistent results. For example, the association between moral courage and satisfaction with the nursing profession remains unclear. There was, however, a clear association between the assessments of moral courage and the confidence to implement care based on ethical principles. Thus, in nursing education, the teaching of the principles remains important.^5^

Managers’ and patients’ views were used to form a comprehensive assessment of GNSs’ moral courage, and their views can be explained in different ways. The managers’ assessments may reflect their longer experience in nursing and their knowledge and experience of nursing ethics in practice.^2,3,60^ It may also be that the GNSs had idealistic views, feeling the need to be patients’ advocates^12–15^ and wanting to be morally courageous, consequently assessing their moral courage as higher than that rated by the managers or evidenced in previous research.^9^ An important insight gained in this study is that the patients assessed the GNSs’ moral courage highest in nearly all participating countries, indicating their positive experiences with students. Although full matching of individual GNSs, patients and their evaluations was not feasible, patients were guided to direct their attention to certain GNS(s) providing care to them. Thus, patients also had a possibility to detect morally courageous action – or lack thereof – while receiving care from GNSs if such situations emerged. However, patients can have conflicting feelings about giving feedback of students; some find it natural whereas others are hesitant.^64^ Nevertheless, as the majority of patients were able to assess GNSs’ moral courage, as an educational implication, patients’ greater contributions for the assessment of students during clinical placements can be encouraged. Patients can also enhance students’ understanding of the possible ethical conflicts in nursing practice from their perspective and the expectations patients have for nurses in this respect.^37,64^

### Limitations

There are limitations in this study. The first one has to do with the international comparison, which is a challenge due to different educational systems despite the common European educational directives.^6^ However, all these GNSs are in the transition period from student to qualified nurse and are thus comparable. Furthermore, the European labour market is free for all of them, providing a reason to carry out comparative studies – even if it is challenging.

The convenience sampling method also involves limitations. However, a power analysis was used to estimate a large enough sample size to ensure the probability that a significant effect is revealed through statistical testing when a true effect really exists. In addition, a common protocol for data collection was used, ensuring the likelihood of representative samples as well. It was not possible to randomise the samples in the countries in terms of the individual background of respondents, for example. Overall, the GNS sample corresponds rather well to the population. For instance, the percentage of practising female nurses in the European region is 84%,^65^ while in this study it was 88%. The total sample in this study also corresponds to another recent European study surveying GNSs in terms of gender and age.^66^

As for the instrumentation of the study, this was the first time a single question derived from the NMCS was used. This was based on earlier study^11^ where overall assessment from this single question was aligned with the results about moral courage from other sections of the scale. With this question, a rough overall assessment of moral courage can be gained, which is justifiable in extensive studies of this kind. However, the use of the entire NMCS is recommended when a more detailed understanding of moral courage is required.

### Implications for further research

There are several implications for further research. First, it would be important to analyse the level of moral courage with different methods. In this study, data-source triangulation was used^40,41^ to form a comprehensive view of GNSs’ moral courage indicating differences between the populations. To analyse further the differences found, matched group design can be considered, for example; however, this was not possible in the current study.

Second, the factors associated with GNSs’ moral courage require further analysis. This is true especially for associations between moral courage and ethics education, clinical practicums and the role of clinical supervisors. Some associations were found, giving ideas for future research. However, it would be important to analyse more multidimensional factors, especially possible supporting factors.

Third, there is a need for a deeper international analysis of GNSs’ moral courage to further explore the reasons behind the variations in GNSs’ moral courage between different countries. In this study, specific cultural factors associated with moral courage were not found due to the instrumentation used.

Finally, there is a need for analysing cases from different databases and registers concerning patient injuries and complaints to gain an understanding of situations where moral courage has emerged.

## Conclusion

In all participating countries, the GNSs self-assessed their moral courage to be rather high, while the managers assessed it lower and the patients higher than the GNS themselves. The associated factors identified may support further strengthening of nursing students’ moral courage. Based on the results, GNSs seem to have confidence and moral courage for ethical situations. In future, there is a need both for studies using multidimensional instruments and for further conceptual analysis of moral courage.
